# Sex-dependent relationships between PFAS and placental transcriptomics identified by weighted gene co-expression analysis

**DOI:** 10.1016/j.envres.2025.122745

**Published:** 2025-09-01

**Authors:** Cynthia Perez, Kyle Campbell, Dana Boyd Barr, Kartik Shankar, Clark Sims, Kevin J. Pearson, Aline Andres, Todd M. Everson

**Affiliations:** a Gangarosa Department of Environmental Health, Emory University Rollins School of Public Health, Atlanta, GA, USA; b USDA Agricultural Research Services, Responsive Agricultural Food Systems Research Unit, College Station, TX, USA; c Arkansas Children’s Nutrition Center, Little Rock, AR, USA; d Department of Pharmacology & Nutritional Sciences, University of Kentucky College of Medicine, Lexington, KY, USA; e Department of Pediatrics, University of Arkansas for Medical Sciences, Little Rock, AR, USA; f Department of Epidemiology, Emory University Rollins School of Public Health, Atlanta, GA, USA

## Abstract

**Background:**

Per- and polyfluoroalkyl substances (PFAS) are environmental toxicants associated with adverse neonatal outcomes. The exact mechanisms by which PFAS impairs neonatal health are undefined, but the placenta is a likely target.

**Objective:**

We applied a systems biology approach to identify placental RNA co-expression modules (gene sets) associated with PFAS exposure and birth weight.

**Methods:**

Placental tissue samples (n = 147) from the GLOWING study underwent RNA-sequencing, and PFAS concentrations were quantified using liquid chromatography-tandem mass spectrometry. We constructed a weighted gene co-expression network using Spearman correlations across 15,028 transcripts, identifying 20 gene modules. Linear regression models were used to examine associations between PFAS and module eigengenes, adjusting for potential confounders. Effect modification by fetal sex was also tested.

**Results:**

One module showed a negative association with perfluorononanoic acid (PFNA; β = −0.012, q = 0.009). This association was sex-specific, with the sexes exhibiting varied PFAS associations but similar directional effects. Genes within the PFNA-associated module were involved in histone modification (q ≤ 0.05) and were enriched for targets of the Vitamin D Receptor (VDR), a transcription factor previously linked to PFAS.

## 1. Introduction

Per- and polyfluoroalkyl substances (PFAS) are characterized by a repetitive chain of carbon and fluorine bonds, along with a distinctive functional group. ((ITRC) ITaRC, 2020) This structural composition renders PFAS a practical choice for repelling water and grease from a wide range of products. This same structure also contributes to its extended half-life and persistent environmental contamination. (Dickman and Aga, 2022; Bonato et al., 2020) Heightened exposure to PFAS can present health risks such as thyroid dysregulation, neurological impairment, and increased susceptibility to cancer. (Mariussen, 2012; Kim et al., 2018; Vieira et al., 2013; Bonefeld-Jorgensen et al., 2014; Brown-Leung and Cannon, 2022) In an effort to mitigate these risks, the U.S. Environmental Protection Agency reached an agreement with eight U.S. companies to cease the use and production of PFAS with the lengthiest history of use, specifically perfluorooctanoic acid (PFOA) and perfluorooctanesulfonic acid (PFOS). (Gold and Wagner, 2020) However, the agreement did not address the replacement PFAS the companies would use, nor did it address remediation of the present environmental contamination of PFOA and PFOS. PFAS are a critical environmental health concern, with exposure being especially concerning during early human development.

Our group and others have shown that PFAS accumulate in placental tissue during pregnancy, and that prenatal exposure to PFAS is associated with molecular responses, low birth weight, impaired cardiovascular health, and increased dyslipidemia in neonates. (Everson et al., 2024; Groisman et al., 2023; Hall et al., 2022) The toxicity of PFAS to the placenta may be due to alterations to lipid metabolism, hormonal imbalance, oxidative stress, and alterations in histone-modifying enzymes. (Abdulkadir et al.) One molecular mechanism of increasing interest involves peroxisome proliferator-activated receptors (PPARs), a nuclear receptor superfamily crucial for embryo implantation and development of the placenta. (Guo et al., 2022) PPARs can regulate the expression of genes essential for cell differentiation and various metabolic processes like glucose homeostasis. (Guo et al., 2022) Research suggests all three PPAR isomers are affected by PFAS, with PPAR *α* being a particularly common target. (Szilagyi et al., 2020) Other nuclear receptors, including androstane receptor and pregnane X, are also impacted by PFAS. (Bjork et al., 2011; Ren et al., 2009) The relationship between PFAS and the activity of key transcription factors, like nuclear receptors, may influence the expression of a plethora of genes. Therefore, it is advantageous to utilize a systems biology approach to better understand impacts of PFAS on transcriptional dysregulation.

Weighted gene co-expression network analysis can model complex interactions between expressed genes and discover emergent system properties. (Langfelder and Horvath, 2008) These networks utilize global gene expression measurements (RNA sequencing or microarray-based data) to reveal sets of genes which may share the same transcriptional regulatory system, members of the same biological pathways, or that are functionally related. (Gaiteri et al., 2014) Weighted gene co-expression networks may be useful in characterizing system-wide placental biological activity and/or disruptions caused by PFAS, providing novel insights into how PFAS affect placental function.

Our previous research indicated that placental PFAS influenced DNA methylation, accelerated gestational age in females, and correlated with a reduced predicted syncytiotrophoblast proportion in males. (Perez et al., 2025) Consequently, we aimed to uncover functional gene networks impacted by PFAS. (Perez et al., 2025; Everson et al., 2025) We applied Weighted Gene Correlation Network Analysis (WGCNA) to identify PFAS-associated co-expressed gene sets (or modules) from placental RNA sequencing profiles. Emerging research, including our own, suggests that in utero exposure to PFAS may lead to sex specific developmental effects in vivo. (Perez et al., 2025; Crute et al., 2023; Negri et al., 2017) Therefore, we hypothesized that PFAS-associated changes in gene expression may be sexually dimorphic. Due to regions of high sequence similarity between the X and Y chromosomes, we employed a sex complement alignment method to identify true sex differences. (Olney et al., 2020) We found one module composed of mainly sex-linked genes to be associated with perfluorononanoic acid (PFNA). Most PFAS do not appear to affect system-wide gene expression in the placenta, but PFNA does only in a sex specific manner.

## 2. Methods

### 2.1. Study population

The Glowing Cohort was established as part of the VICTER consortium project (R01 ES032176) to investigate the effects of in utero PFAS exposure on placental omics. Between 2010 and 2014, 300 pregnant participants were recruited from the Little Rock, Arkansas, metropolitan area. Further details on recruitment criteria can be found in prior work. (Perez et al., 2025; Everson et al., 2025) In short, the cohort consists exclusively of non-smoking participants over the age of 21 without complicated births or serious medical conditions. At enrollment, participants were categorized as lean or overweight based on pre-pregnancy BMI, which was determined by self-reported height and weight.

### 2.2. Placenta collection and quantification of PFAS

A subsample of 150 participants provided sufficient placental tissue for RNA sequencing and PFAS quantification. Placental physical properties (e.g., weight) were measured within 2 h of collection, after the removal of the umbilical cord and fetal membranes. Approximately 1 g of placental tissue from six random sites within the villous core was pulverized in liquid nitrogen and flash frozen. We have described the quantification of the 17 PFAS chemicals in our population sample via liquid chromatography-tandem mass spectrometry (LC-MS/MS) elsewhere. (Perez et al., 2025; Everson et al., 2025) We restricted our analysis to the 5 most well-detected PFAS with above limit of detection rates greater than 65 % among all samples: perfluorooctane sulfonate (PFOS), perfluorohexane sulfonic acid (PFHxS), perfluorononanoic acid (PFNA), Perfluorooctanoic acid (PFOA), and perfluorodecanoic acid (PFDA). Samples were randomized across runs to reduce potential batch effect. The limit of quantification (LOQ) values for PFAS are represented in Table 1 along with concentration ranges. Values less than the LOQ were replaced with $\frac{\mathrm{LOQ}}{\sqrt{2}}$ to minimize potential bias. (Hornung and Reed, 1990)

### 2.3. RNA sequencing data process

To conduct transcriptomic analyses, RNA was isolated from pulverized heterogenous placenta samples. Total RNA was extracted from the placental samples using TRI reagent, followed by purification using RNeasy mini columns and deoxyribonuclease digestion. Directional RNA-sequencing libraries were created using NebNext Ultra reagents and sequenced on an Illumina HiSeq 4000 platform, generating approximately 30 million reads per sample. All RNA samples were analyzed for purity using A260/A280 ratio and for integrity using Experion Total RNA StdSens reagents. RNA Quality Indicator (RQI) values on all samples were above >8.0. Before and after trimming with Trim Galore, FASTQC and MultiQC were respectively used to assess individual and aggregated sample quality. (Krueger; Ewels et al., 2016; Andrews, 2010) Three samples were excluded because they lacked the yield for both PFAS quantification and RNA sequencing. The trimmed sequences were then aligned to the Gencode GRCh38.p12/hg38 gene annotation using STAR in a sex complement manner where the Y pseudoautosomal regions (PAR 1 and PAR 2) are ignored for males and the Y chromosome is completely ignored for females. (Olney et al., 2020; Dobin et al., 2013) RNA sequencing alignment files were then sorted and indexed using bamtools. (Barnett et al., 2011) Mapped reads were quantified and summarized into counts using featureCounts. (Liao et al., 2014) QC metrics required that samples have more than 12.5M and fewer than 90M sequences after trimming, and less than 30 % of reads that deviate from the normal distribution of per-sequence GC content based on the FastQC report. (Olney et al., 2022) All 150 samples passed QC metrics. The analytic dataset consisted of 15,028 RNA transcripts, including 513 X-linked genes and 16 Y-linked genes. Additionally, to address potential confounding by batch variation effects, we corrected gene expression counts with the ComBat function from the sva package (v 3.52.0), while protecting the covariates in our primary models (maternal age, maternal education, gestational age at delivery, gestational weight gain, biological sex of the neonate, maternal BMI category at enrollment, and the first principal component of RNA-seq data). (Leek et al., 2012)

### 2.4. Statistical analyses

#### 2.4.1. Weighted gene Co-expression network analysis

All analyses were performed using R (version 4.2.2). To identify system-wide gene expression patterns, we used Weighted Gene Co-expression Network Analysis (WGCNA), which aims to discern clusters of genes that display comparable patterns of expression, indicating potential functional relationships among them in the WGCNA R package (v 1.73). (Langfelder and Horvath, 2008) To prepare gene expression data for WGCNA, we normalized expression count data with upper quartile normalization and variance-stabilizing transformation using the R package TRAPR. (Lim et al., 2017) These normalization methods were chosen to optimize co-expression module identification via WGCNA. (Vandenbon, 2022) We chose to employ a signed network approach, which focuses on the strength of positive gene-gene correlations to draw network connections. While there are other network types, signed networks provide ease of biological interpretability as compared to networks that include negative correlations. (Langfelder, 2018)

To construct the signed network, we utilized the upper-quartile normalized, variance-stabilized, and batch-corrected mRNA-seq data to calculate pairwise gene-gene Spearman correlations across 15,028 genes and 147 participants. These correlation values were applied in the following equation to ascertain the similarity, *s*, between genes *i* and *j*: s_ij_ = 0.5 + 0.5*cor(i,j), which quantifies the relationship between genes and penalizes negative correlations. Consequently, negative correlations had fewer connections in the adjacency matrix, a_ij_ = |s_ij_|^β^, where β is a parameter determining the scale-free topology of the network. The adjacency matrix outlines the network’s structure before the clustering process, aimed at establishing co-expression patterns among genes.

To achieve a scale-free topology, we utilized Spearman correlation and selected β = 5 based on the pickSoftThreshold function. We calculated eigengenes, first principal component of each module, to represent overall module expression. The package arbitrarily assigned colors to each identified module. Grey was assigned to genes that did not form a distinct cluster.

#### 2.4.2. Differential expression analysis

To address the right skewed distributions of PFAS measures, PFAS levels were natural log transformed. All models included the following covariates: maternal age (continuous), maternal education (categorical), gestational age at delivery (continuous), gestational weight gain (continuous), biological sex of the neonate (dichotomous), maternal BMI category at enrollment (dichotomous), and the first principal component of RNA-seq data (continuous). These have been previously documented as confounders in studies focused on in utero PFAS exposure. (Hall et al., 2022; Bangma et al., 2020) The first principal component was included in our models to account for RNA-sequencing technical differences or other unmeasured sources of variation (Supplemental Fig. 1).

In addition to WGCNA analyses, we conducted differential expression analyses by empirical Bayes-based models through the limma R package (v 3.60.6) to determine if individual gene expression was associated with PFAS concentration. We assessed the effects of PFAS on all 15,028 genes and additionally honed our analysis to the 519 gonosomal transcripts in sex-stratified models where we dropped Y-linked genes for female specific analysis. P-values were adjusted using Benjamin-Hochberg correction (15,028 tests per PFAS).

#### 2.4.3. Module eigengenes and PFAS analysis

We investigated potential associations between placental PFAS concentrations and changes in the module eigengenes using multiple linear regression. For these models, PFAS concentrations served as the independent variables and the module eigengenes were treated as the dependent variables, while controlling for confounders. We also explored whether there were significant interactions between PFAS and sex on module eigengene levels by including a sex by PFAS cross-product term in our models. To investigate potential effect modification by sex in the association between PFAS and module eigengenes, we then stratified the models by sex. Lastly, we explored the associations between module eigengenes and birth outcomes (gestational age, birth weight, and birth length) to examine whether the same modules impacted by PFAS are also associated with these characteristics. The models were adjusted using the same covariates as those employed in modeling the effects of PFAS on eigengenes and in the differential expression analysis.

Gene set enrichment analysis was performed for each module using the R package gprofiler2 with the database Gene Ontology (GO). (Ashburner et al., 2000; Gene Ontology et al., 2023; Kanehisa and Goto, 2000) The REVIGO tool was used to filter out redundant terms and to visualize enrichment. (Supek et al., 2011) We utilized a placental transcription factor (TF) network to identify possible reasons for co-expression within each module to determine enriched TFs through a Fisher’s exact test. (Paquette et al., 2024) The hub gene was determined using the WGCNA function chooseTopHubinEachModule, which selects genes with the most connections. These secondary analyses aided in understanding the effects of PFAS on module eigengenes.

## 3. Results

### 3.1. Sample description

In our study, 150 placental RNA-sequencing samples passed initial QC. We excluded 2 samples with insufficient yield for PFAS quantification and 1 sample that did not cluster with the reported sex assigned at birth, resulting in a final sample size of N = 147. The demographic characteristics of the study population are summarized in Table 2. Maternal ages ranged from 22 to 42 years old, with a mean age of 30.59 years at the time of birth. The sex distribution of neonates had a greater proportion of male newborns (62.59 %). Participants primarily self-reported their ethnicity and race as non-Hispanic White (80 %).

Among the 17 analyzed PFAS compounds, PFOA, PFHxS, PFNA, PFDA, and PFOS were detectable in more than 65 % of samples and were the focus of this analysis. Over 50 % of samples exceeded the LOQ for all five chemicals, and only one sample had concentrations lower than the LOQ for all five PFAS. Concentrations of these five compounds were moderately correlated (p-values <0.05) with each other, with Spearman correlations ranging from 0.3 to 0.71 (Supplemental Fig. 2). The subsample used in this analysis produced similar demographic and PFAS concentration metrics comparable to those in our previous publications. (Perez et al., 2025; Everson et al., 2025)

### 3.2. Differential expression

**Fig. 1.**
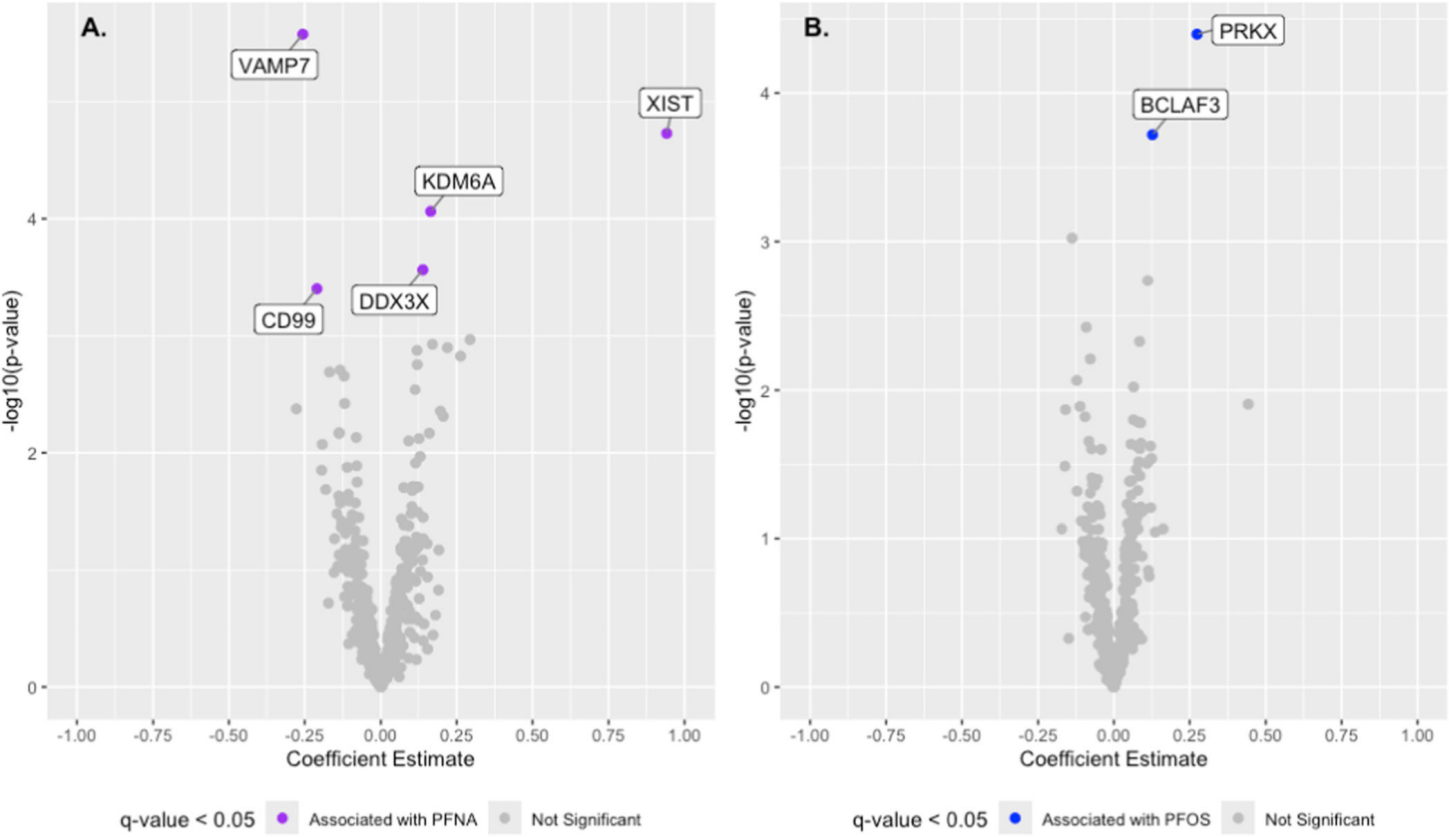
Differential expression analysis in the female strata conducted between 513 X-linked mRNA transcripts, for PFNA (A) and PFOS (B). The x axis is the coefficient estimate in response to PFNA or PFOS. The y-axis is the log_10_ transformed p-value. Significant differentially expressed genes (DEGs) are denoted in purple for PFNA or blue for PFOS. (For interpretation of the references to color in this figure legend, the reader is referred to the Web version of this article.)

We first performed differential gene expression analysis to identify individual genes whose expression is associated with any of the 5 well-detected PFAS. This was achieved through a series of empirical Bayes-based models, where the dependent variable was the expression of one gene, and the independent variable was a PFAS congener and adjusted for covariates. We found no association between PFAS and gene expression after Benjamin-Hochberg correction (Results available here). However, we hypothesized that sex-specific associations among the sex-linked genes may exist. In the female stratum, seven genes were significantly associated with a PFAS chemical (adjusted for 513 tests). Genes *DDX3X* (q-value = 0.035), *KDM6A* (q-value = 0.015), and *XIST* (q-value = 0.005) were positively associated with PFNA while *VAMP7* (q-value = 0.001) and *CD99* (q-value = 0.041) were negatively associated with PFNA (Fig. 1A). The expression of genes *BCLAF3* (q-value = 0.05) and *PRKX* (q-value = 0.021) were positively associated with PFOS (Fig. 1B). In the male stratum, no differential expression was observed.

### 3.3. Placental Gene Co-expression network

Next, we aimed to identify placental gene expression modules and their associated biological functions in our dataset, which could be impacted by PFAS exposure. For this, we used weighted gene co-expression network analysis (WGCNA), which identified 20 co-expressed modules, and excluded grey which contained 214 genes that were not members of a co-expressed module (Supplemental Fig. 3). The number of genes within each module ranged from 37 to 8328. The proportion of variance explained by the eigengenes, the first principal component of each module, ranged from 31.1 % (brown) to 94.2 % (light green) with an average proportion of 53.9 %.

**Fig. 2.**
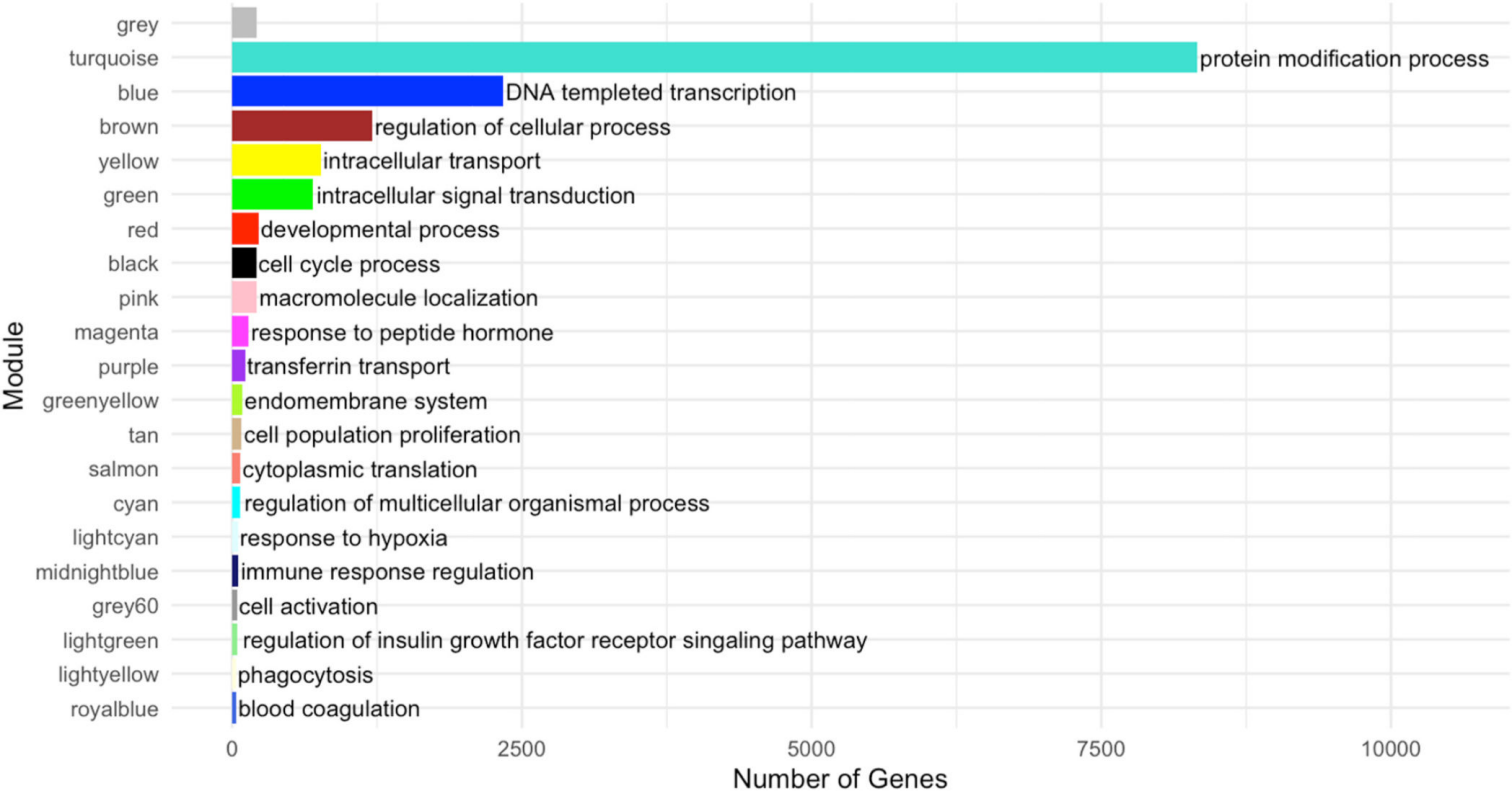
Weighted gene co-expression network analysis identified 20 co-expressed modules, and each was assigned a color (y-axis). The number of genes in each of the modules (x-axis) ranged from 37 to 8328 genes. We labeled each module with the most pertinent biological processes provided by REVIGO. Grey includes 214 genes not co-expressed. (For interpretation of the references to color in this figure legend, the reader is referred to the Web version of this article.)

Gene set enrichment analysis was performed on each module separately, using the GO consortium (Supplemental File 1). (Ashburner et al., 2000) Each module exhibited enrichment for at least one GO term within one of the three biological domains: molecular function, biological process, or cellular compartment. Module-specific enrichment yielded multiple redundant terms that were then summarized and represented as tree maps by REVIGO. For simplicity, we labeled each module with the most pertinent biological process chosen from the REVIGO function Tree Map that clustered the most GO terms and was the most significant (Fig. 2). (Supek et al., 2011)

We conducted a transcription factor (TF) enrichment analysis using a TF network specific to the placenta to explore the reasons for coexpression within the modules. (Paquette et al., 2024) TFs that targeted genes within a module were assigned an enrichment p-value and q-value for each TF (Supplemental File 2). The enriched TFs were not significant after adjustment for the blue and green modules. The magenta module had the highest number of enriched TFs (q-value <0.05) with 39, while the light green module had the fewest, with only 2.

We also assessed whether any module eigengenes were related to birth outcomes. We modeled these relationships using linear regression analysis and adjusted for confounders. The module eigengenes of tan and green yellow were associated with birth weight, p-value <0.05. The module eigengenes magenta, midnight blue, and tan were related to gestational age, p-value <0.05. However, none of these associations were significant after correction for multiple testing (all q > 0.05). (Supplemental File 3).

### 3.4. Module and PFAS associations

We individually modeled the relationship between each of the 20 co-expressed module eigengenes and each of the 5 PFAS, adjusted for confounders. After false discovery rate adjustment, one eigengene, light green, was significantly associated with PFNA (β = −0.012, q-value = 0.009). This module contains 40 genes, of which 33 are sex-linked (17 X-linked and 16 Y-linked). Chromosome 20 is the only autosome with more than one gene represented within the light green module (Table 3). Interestingly, consistent with our sex-specific differential expression analysis, the light green genes *KDM6A*, *PRKX*, and *XIST* were also significantly differentially expressed among females.

Light green contained one known nuclear hormone receptor, VDR. We found that other nuclear hormone receptors of interest, PPARs α and δ, are in the brown module, and PPAR γ is in the turquoise module. Both modules have an unadjusted p-value <0.05 with PFDA, but the association does not remain significant after correcting for multiple testing, with q-values of 0.263.

### 3.5. Investigating sexual dimorphism of PFAS associations

Given that many genes in light green were located on sex chromosomes, and PFAS are hypothesized to have sex-specific effects, we investigated whether there were statistical interactions in linear models between sex and PFAS exposure on each of the eigengenes. We identified a significant interaction between PFNA and sex for the light green eigengene (β = 0.023, q-value = 0.01, Table 4). Several other PFAS exhibited nominally significant (raw p < 0.05) interactions for light green.

We then characterized sex-specific relationships via sex-stratified linear regression analysis. We observed consistent directionality across sex strata, where eigengene levels decreased in association with almost all PFAS, but that these effects were stronger amongst female placentas, particularly for PFNA (β = −0.03, p-value = 0.0001) (Table 5). We explored whether PFAS concentrations themselves differed between the sexes via *t*-test comparisons, but none of the 5 PFAS compounds differed significantly between male and female placenta. Together, these results suggested that sexually dimorphic expression in light green was not attributable to PFAS concentration differences and may be due to sex-specific effect modification.

### 3.6. Characterizing the PFAS-associated module – light green

**Fig. 3.**
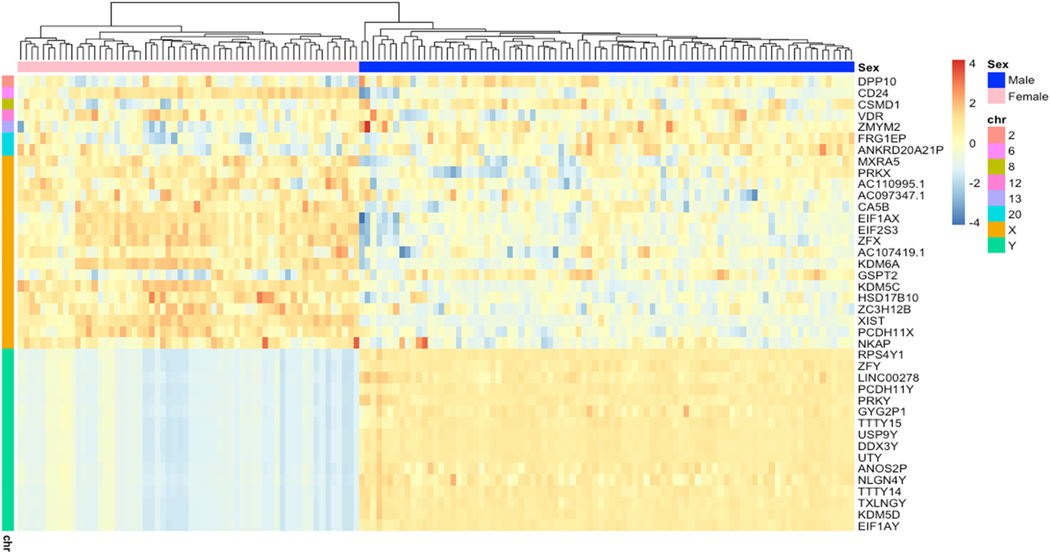
Heat map of expression profiles of light green genes for each participant distinguished by sex and chromosome. (For interpretation of the references to color in this figure legend, the reader is referred to the Web version of this article.)

**Fig. 4.**
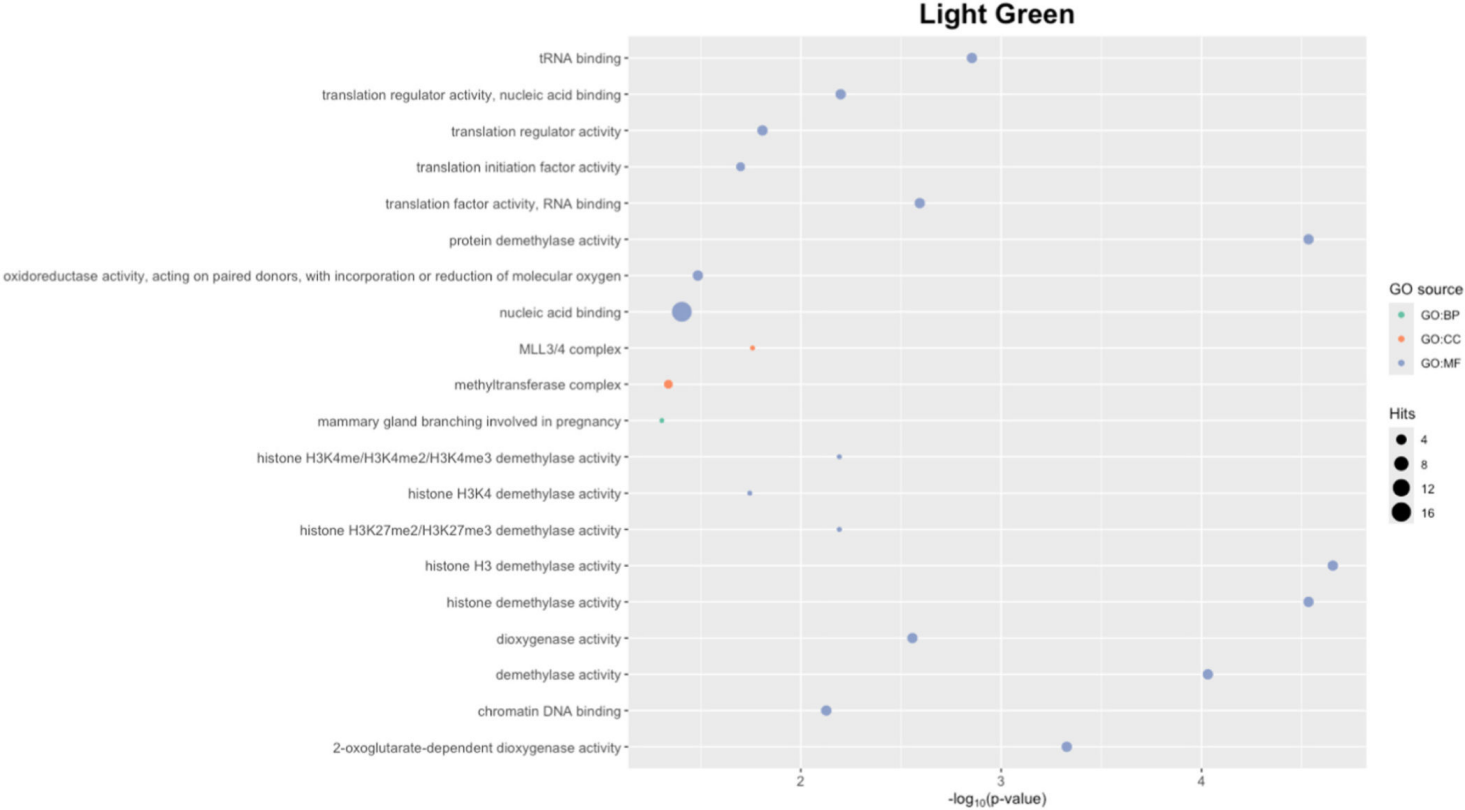
The significant GO terms for light green module are mainly representative of histone activity. The circles indicate the number of genes/hits corresponding to the term and the color relates to the GO source. (For interpretation of the references to color in this figure legend, the reader is referred to the Web version of this article.)

Last, we aimed to characterize the expression patterns, GO terms, and upstream TFs that are important to the only PFAS-associated module, light green. T-test showed that males clearly had a significantly higher eigengene levels compared to females (p = 2.2e-16) and there was a distinct separation in individual gene expression patterns between the sexes in an expression heatmap (Fig. 3). Interestingly, while clear sex-specific expression patterns were expected and observed for sex-linked genes, the sex differences in expression also extended to the autosomal genes featured in light green. The GO terms associated with light green were largely representative of histone modification processes (Fig. 4). When using REVIGO to summarize redundant terms affiliated with biological processes, this module was assigned regulation of insulin growth factor receptor signaling pathway (p-value <0.05). Because TFs can have broad effects on gene activity and may provide insights into co-expression patterns, we identified TFs that are linked to the expression of the genes in the light green module. Placental TF enrichment analysis identified 2 TFs with overrepresented gene-targets in light green (q-values <0.05) (Table 6). *VDR* targeted 5 light green genes (*KDM5C*, *KDM6A*, *UTY*, *EIF1AX*, *EIF2S3*), while *ZFX* targeted a total of 6 genes (*KDM5C*, *KDM6A*, *UTY*, *EIF1AX*, *EIF2S3*, *VDR*). The hub gene, *USP9Y*, was not a target of the enriched TFs.

## 4. Discussion

In this study, we applied WGCNA to build a gene network using placental RNA-sequencing data to identify gene expression patterns linked to PFAS exposure. Our analysis revealed a PFAS-related module termed “light green” that was characterized predominately by sex-specific genes and enriched for genes related to histone modification and tissue-specific gene targets of the transcription factors *VDR* and *ZFX*. Prior applications of WGCNA to human placental RNA sequencing data have uncovered modules associated with various birth outcomes and environmental exposures. (Deyssenroth et al., 2017, 2021) While other co-expression studies have identified sex-specific modules, these prior studies did not identify sex-specific associations with exposure and outcomes. (Deyssenroth et al., 2017, 2021; Zhu et al., 2023; Rey et al., 2021) The sex-related modules identified in prior research showed enrichment for biological processes involving DNA conformational changes and post-transcriptional modifications. (Deyssenroth et al., 2017, 2021; Zhu et al., 2023; Rey et al., 2021) Our sex-specific light green module was also enriched for some of these molecular processes.

Notably, several terms related to histone modifications were overrepresented within light green. Histone modifications can switch chromatin structure between condensed and relaxed states, influencing DNA conformation and gene expression. (Tricarico et al., 2020) Early research suggests PFAS exposure can alter histone acetylation and methylation and may directly interact with enzymes like histone acetyltransferases (HATs) and deacetylases (HDACs). (Abdulkadir et al.) The liver of female mouse pups from mothers exposed to PFOA exhibited reduced HAT activity and increased HDAC activity. (Li et al.a) Cord blood PFAS levels strongly correlated with H3K43 levels measured from blood leukocytes of 2-year-old children and had different directional effects. (Tsai et al.) Both studies suggest that histone modifications are a mechanism of in utero PFAS toxicity.

In addition to histone modification, we noted post-transcriptional modifications as a recurring theme in the enriched GO terms for light green. Known post-translational modifications associated with PFAS include long non-coding RNAs and microRNAs. (Abdulkadir et al.) We identified three long non-coding RNAs in the light green module. Research on the relationship between PFAS and long non-coding RNAs is limited but may contribute to immune function dysregulation. (Li et al. b) The light green module illustrates previous analyses of potential mechanisms behind PFAS toxicity, with sex differences influencing tissue-specific transcriptional regulation. (Olney et al., 2022; Blencowe et al., 2022)

Most of the light green genes identified in our investigation are involved in gene expression regulation and have known X-Y homologs. (Bellott et al., 2014) The X-Y pairs in light green include: *PRKY/PRKX, KDM6A(UTX)/UTY*, *KDM5C/KDM5D*, *EIFAX/EIF1AY*, and *ZFX/ZFY*. (Bellott et al., 2014) These X homologs are not subject to X-inactivation, leading to higher expression in females. X-inactivation, vital for balancing gene dosage between sexes in mammals, is influenced by factors such as the *XIST* gene, expressed from the inactive X chromosome and present in light green. (Berletch et al., 2010; Carrel and Willard, 2005) Genes active on the ‘silent’ X chromosome are thought to contribute to sex differences and are particularly significant for brain function. (Xu and Disteche, 2006)

While most studies of prenatal PFAS exposure have not focused on sex-specific effects, several studies have found males and females may be differentially impacted by PFAS. For instance, neurobehavioral disorders can differ in severity by sex and have been associated with PFAS. (Braun, 2017) Specifically, PFOS and PFOA demonstrate neurotoxic traits, although the connection between PFAS and neurodevelopment remains unclear. (Brown-Leung and Cannon, 2022; Starnes et al., 2022) However, there is more consistent evidence linking PFAS exposure to cardiovascular diseases in adulthood. Emerging research suggests that genes on the inactivated X chromosome might also affect the cardiovascular system. (Aguado et al., 2022; Lau et al., 2022) While our study does not link X-Y paralogs and their expression with disease, we identified a statistically significant interaction between PFNA exposure and sex for the light green module eigengene, which indicates that the biological sex of placenta could be key in understanding how PFNA contributes to developmental outcomes. This is especially important considering that sex differences in gene expression apparent in early and term placentas are correlated with sex differences in adult tissues, specifically in the brain. (Olney et al., 2022; Rosenfeld, 2021)

Although emerging evidence supports that PFAS may have several sex-specific effects, investigations exploring the effects of PFAS exposure on sex-linked gene expression remain limited. To our knowledge, *DDX3Y* is the only light green sex-linked gene with previously reported PFAS associations. *DDX3Y* was upregulated in the livers of male mice fed a high fat diet and exposed to PFNA. (Pfohl et al., 2021) Research into the association between gonosomal genes and PFAS could be limited because of over representation of males in biomedical research studies. (Plevkova et al., 2020) However, many research studies have speculated that PFAS can impact the function of sex chromosomes because PFAS can impair fertility, menstruation, and spermatogenesis. (Song et al., 2018; Upson et al., 2022; Wang et al., 2023) The light green hub gene, *USP9Y*, is involved in male fertility and spermatogenesis and may be key to understanding how PFAS disrupts these processes. (Colaco and Modi, 2018) Sex-linked genes ultimately modulate all sex related processes; however, there is no research suggesting that PFAS can alter the expression of these genes directly.

While the light green module is composed primarily of X-linked and Y-linked genes, there are also a handful of autosomal genes, most notably the gene for the vitamin D receptor, *VDR. VDR* is the only light green gene previously associated with PFAS exposure. In silico and in vitro assays have shown that PFNA binds to *VDR* and changes the activity of VDR’s target genes. (Tachachartvanich et al., 2022; Di Nisio et al., 2020) Additionally, a recent large study of adults showed that increasing levels of PFAS exposure were associated with decreased serum levels of vitamin D, but only among females. (Zhao et al., 2024) Vitamin D insufficiency has also previously been shown to decrease fetal growth. (Nguyen et al., 2018) In our study, *VDR* expression itself was not associated with any individual PFAS, but *VDR* was within the light green module along with several of its target genes (*KDM5C*, *KDM6A*, *UTY*, *EIF1AX*, *EIF2S3*). Importantly, *VDR* is a transcription factor that mediates the actions of 1,25(OH)_2_D_3_ (the biologically active form of vitamin D), driving transcriptional responses in several tissues, including the placenta. (Shin et al., 2010) VDR is also a nuclear hormone receptor; it can trigger transcriptional changes after ligand-induced activation. In silico analysis identified 14 PFAS chemicals, which included PFNA, that were predicted to strongly interact with VDR, similarly to natural ligands. (Azhagiya Singam et al., 2023) Although light green did not contain PPARs, which are primary molecular targets of PFAS, two modules containing PPARs (α, δ, and γ) were nominally associated with increasing placental PFDA levels. Others have shown PPARα to be responsive to PFDA and the PPAR family of receptors are generally implicated as PFAS targets. (Szilagyi et al., 2020; Luo et al.) Overall, our findings support that nuclear hormone receptors are a plausible mechanism of placental PFAS toxicity. Additionally, our study contributes to the growing body of evidence that the molecular mechanisms involved in vitamin D metabolism are potential targets of PFAS, and these responses may affect male and female placentas differently.

This investigation reports innovative findings about sex-specific placental transcriptional responses to PFAS, but these findings should be interpreted within the context of some limitations. We identified some autosomal gene expression associations with PFAS, but these did not pass significance thresholds that were corrected for multiple testing, indicating a potential lack of power to detect small effect sizes exacerbated by the necessity of sex stratification to study sex-specific effects. The results do not consider placental cell heterogeneity, and thus our approach may have missed cell-type specific responses to PFAS (Mai et al.) which is a limitation of our study. The rapidly advancing field of single-cell and single-nucleus omics can help to answer this question in future studies. (Kong et al.) The demographic composition of our study sample may limit the generalizability of our results, and since placental samples were collected between 2010 and 2014, we did not detect high enough concentrations to study the impact of more recently emergent PFAS chemicals. Nonetheless, this cohort is still one of the only to quantify PFAS in placenta with composite birth outcome and molecular omics data. We showcase that even healthy pregnancies can be affected by in utero exposure to PFAS.

The association of the light green module with PFNA suggests that PFNA contributes to sexual dimorphic responses by interacting with sex-linked genes. Light green genes were found to contain X-Y paralog pairs and genes crucial for transcriptional regulation known to influence reproduction, cardiovascular health, and neurodevelopment. The module also was enriched for the TF *VDR,* a gene represented within light green that has also been previously associated with PFAS exposure. Overall, our study provides novel findings that higher levels of placental PFNA were associated with downregulation of some placental genes, but only among female placentas. These results were consistent with our sex-stratified differential expression analysis where only females exhibited a response. Some of the light green genes are targets of *VDR*, implicating changes to vitamin D metabolism and downstream pathways, which may be related to the genes within the module that are broadly involved in insulin growth factor receptor signaling and histone modifications. Further research is needed to understand whether these gene expression responses to PFAS are related to placental function, pregnancy outcomes, and children’s health outcomes.

## 5. Conclusion

Our research indicates that prenatal exposure to PFNA influences placental gene expression differently based on sex, which may affect insulin growth factor signaling and histone modification. The presence of VDR in this module and the transcription enrichment analysis align with previous findings regarding PFAS and VDR interactions. This module related to PFNA could shed light on the molecular pathways connecting PFAS exposure to health outcomes in neonates.

## Supplementary Material

1

2

3

4

5

6

## Figures and Tables

**Table 1:** Summary statistics of 17 PFAS chemicals measured in N = 147 RNA-sequencing profiles with sufficient yield for PFAS quantification. The limit of qualification (LOQ) is the lowest concentration of a substance reliably measured with acceptable accuracy and precision.

PFAS	Geometric Mean	Geometric SD	LOQ Range	>LOQ (%)

PFOS	0.375	2.010	0.021–2.038	99 %
PFNA	0.049	1.866	0.007–0.222	98 %
PFHxS	0.047	2.795	0.007–0.816	83 %
PFOA	0.051	2.890	0.012–0.416	72 %
PFDA	0.025	2.461	0.007–0.108	71 %
PFUnDA	0.020	2.168	0.013–0.138	31 %
PFHpA	0.007	1.816	0.006–0.184	20 %
HFPO-DA	0.015	1.495	0.014–0.420	4 %
PFHxA	0.007	1.241	0.007–0.237	2 %
MePFOSAA	0.073	1.240	0.071–0.347	2 %
PFBS	0.007	1.151	0.007–0.039	1 %
PFDoDA	0.009	1.143	0.009–0.032	1 %
PFOSA	0.071	1.061	0.071–0.145	1 %
PFPeA	0.014	1.000	0.014–0.014	0 %
PFHpS	0.014	1.000	0.141–0.141	0 %
PFDS	0.035	1.000	0.035–0.035	0 %
EtPFOSAA	0.071	1.000	0.071–0.071	0 %

**Table 2:** Demographic characteristics of N = 147 participants.

Maternal and Infant Characteristics	Mean (SD)

Gestational Weight Gain (kg)	11.86 (4.22)
Gestational Age (weeks)	39.33 (0.84)
Maternal Age (years)	30.52 (3.46)
Birth Weight (kg)	3.54 (0.43)
Birth Length (cm)	51.28 (2.31)
	N (%)
BMI Enrollment Category	
Mother with normal weight	73 (50)
Mother with overweight or obesity	74 (50)
Sex of Infant	
Female	60 (41)
Male	87 (59)
Maternal Education	
College Graduate	56 (38)
Graduate Training or Degree	44 (30)
High School Graduate, GED, Associate, Partial College, or Specialized Training	47 (32)
Race of Infant	
White	121 (82)
Non-White	26 (18)
Ethnicity of Infant	
Hispanic	5 (3)
Non-Hispanic	142 (97)

**Table 3:** The 40 light green genes featuring their chromosome location. The module includes 33 sex linked genes (17 X-linked and 16 Y-linked). Gene annotation file included long coding RNAs with no official name as identified by the HUGO gene name nomenclature committee.

Chromosome	Gene Symbol	Full Name

2	*DPP10*	Dipeptidyl Peptidase Like 10
6	*CD24*	Cd24 Molecule
8	*CSMD1*	Cub And Sushi Multiple Domains 1
12	*VDR*	Vitamin D Receptor
13	*ZMYM2*	Zinc Finger Mym-Type Containing 2
20	*FRG1EP*	Fshd Region Gene 1 Family Member E, Pseudogene
20	*ANKRD20A21P*	Ankyrin Repeat Domain 20 Family Member A21, Pseudogene
X	*MXRA5*	Matrix Remodeling Associated 5
X	*PRKX*	Protein Kinase Camp-Dependent X-Linked Catalytic Subunit
X	*AC110995.1*	Long Intergenic Non-Protein Coding Rna 3070
X	*AC097347.1*	
X	*CA5B*	Carbonic Anhydrase 5B
X	*EIF1AX*	Eukaryotic Translation Initiation Factor 1A X-Linked
X	*EIF2S3*	Eukaryotic Translation Initiation Factor 2 Subunit Gamma
X	*ZFX*	Zinc Finger Protein X-Linked
X	*AC107419.1*	
X	*KDM6A*	Lysine Demethylase 6A
X	*GSPT2*	G1 To S Phase Transition 2
X	*KDM5C*	Lysine Demethylase 5C
X	*HSD17B10*	Hydroxysteroid 17-Beta Dehydrogenase 10
X	*ZC3H12B*	Zinc Finger Ccch-Type Containing 12B
X	*XIST*	X Inactive Specific Transcript
X	*PCDH11X*	Protocadherin 11 X-linked
X	*NKAP*	Nfkb Activating Protein
Y	*RPS4Y1*	Ribosomal Protein S4 Y-Linked 1
Y	*ZFY*	Zinc Finger Protein Y-Linked
Y	*LINC00278*	Long Intergenic Non-Protein Coding Rna 278
Y	*PCDH11Y*	Protocadherin 11 Y-Linked
Y	*PRKY*	Protein Kinase Y-Linked
Y	*GYG2P1*	Glycogenin 2 Pseudogene 1
Y	*TTTY15*	Testis Expressed Transcript, Y-Linked 15
Y	*USP9Y*	Ubiquitin Specific Peptidase 9 Y-Linked
Y	*DDX3Y*	Dead-Box Helicase 3 Y-Linked
Y	*UTY*	Ubiquitously Transcribed Tetratricopeptide Repeat Containing, Y-Linked
Y	*ANOS2P*	Anosmin 2, Pseudogene
Y	*NLGN4Y*	Neuroligin 4 Y-Linked
Y	*TTTY14*	Testis Expressed Transcript, Y-Linked 14
Y	*TXLNGY*	Taxilin Gamma Y-Linked (Pseudogene)
Y	*KDM5D*	Lysine Demethylase 5D
Y	*EIF1AY*	Eukaryotic Translation Initiation Factor 1A Y-Linked

**Table 4:** Linear regression analyses examining PFAS as the independent variable and the eigengene of the light green module as the dependent variable (adjusted for confounders), both with and without an interaction term between a PFAS chemical and the biological sex of neonate.

Main Effect				
PFAS	95 % CI	Estimate	p-value	q-value

PFOA	[−0.005,0.002]	−0.001	0.465	0.952
PFHxS	[−0.004, 0.004]	−0.0002	0.921	0.994
PFNA	[−0.017, −0.006]	−0.012	<0.001	0.009
PFDA	[−0.009, −0.001]	−0.005	0.013	0.263
PFOS	[−0.012, −0.001]	−0.006	0.019	0.263

Interaction				
PFAS	95 % CI	Estimate	p-value	q-value

PFOA	[−0.010, 0.001]	0.0062	0.080	0.43
PFHxS	[−0.006, 0.006]	−0.0005	0.894	0.946
PFNA	[0.011, 0.035]	0.0227	<0.001	0.01
PFDA	[−0.014, −0.0015]	0.0048	0.264	0.596
PFOS	[−0.019,−0.005]	0.0111	0.036	0.395

**Table 5:** Sex stratified linear regression analyses examining PFAS as an independent variable and eigengene of the light green module as a dependent variable (adjusted for confounders).

Male			
PFAS	95 % CI	Estimate	p-value

PFOA	[−0.002, 0.004]	0.001	0.423
PFHxS	[−0.003, 0.002]	0.000	0.860
PFNA	[−0.008, −0.0003]	−0.004	0.045
PFDA	[−0.006, −0.0001]	−0.003	0.029
PFOS	[−0.005, 0.003]	−0.001	0.536

Female			
PFAS	95 % CI	Estimate	p-value

PFOA	[−0.012, 0.002]	−0.005	0.174
PFHxS	[−0.009, 0.009]	0.0001	0.982
PFNA	[−0.038,−0.013]	−0.026	<0.001
PFDA	[−0.018,0.001]	−0.008	0.092
PFOS	[−0.022, −0.0004]	−0.011	0.042

**Table 6:** Light green enriched placenta specific transcription factors identified using fisher exact test and adjusted for multiple testing correction.

TF	p-value	q-value	Light Green Gene Targets

VDR	1.76E-07	3.52E-07	*KDM5C*, *KDM6A*, *UTY*, *EIF1AX*, *EIF2S3*
ZFX	4.57E-06	4.57E-06	*KDM5C*, *KDM6A*, *UTY*, *EIF1AX*, *EIF2S3*, *VDR*

## Data Availability

The data that support the findings of this study are available from the corresponding author upon reasonable request. Scripts are available at https://github.com/cmperez19/PFAS_Placenta_RNAseq_WGCNA.

## Appendix A. Supplementary data

Supplementary data to this article can be found online at https://doi.org/10.1016/j.envres.2025.122745.
