# Supplementary figures and images for: Sex-dependent relationships between PFAS and placental transcriptomics identified by weighted gene co-expression analysis
Source: Environ Res. Author manuscript; Available in PMC 2025 Dec 21. (PMC12718636; doi:10.1016/j.envres.2025.122745)

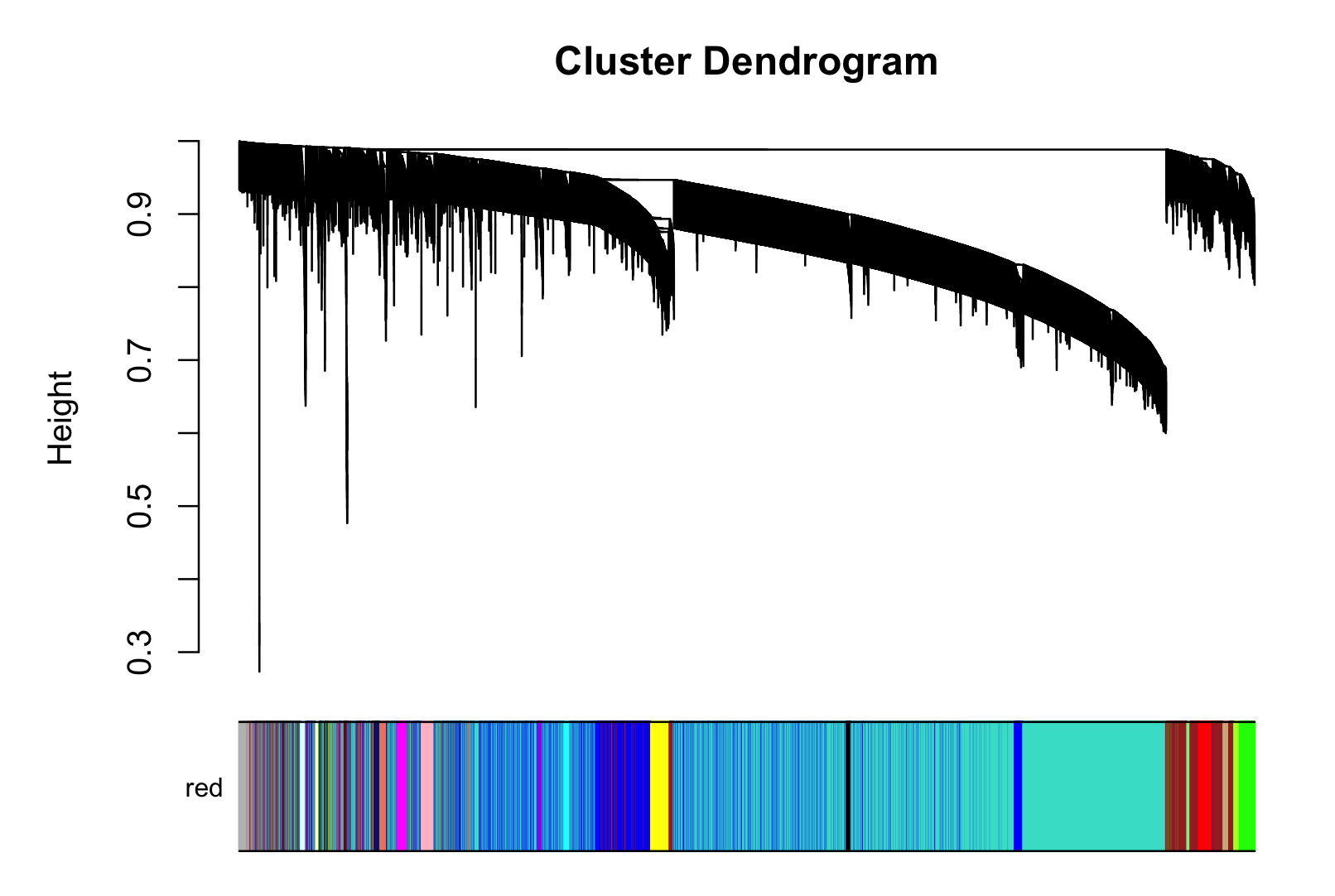

Supplement: 3 [file NIHMS2109826-supplement-3.png]

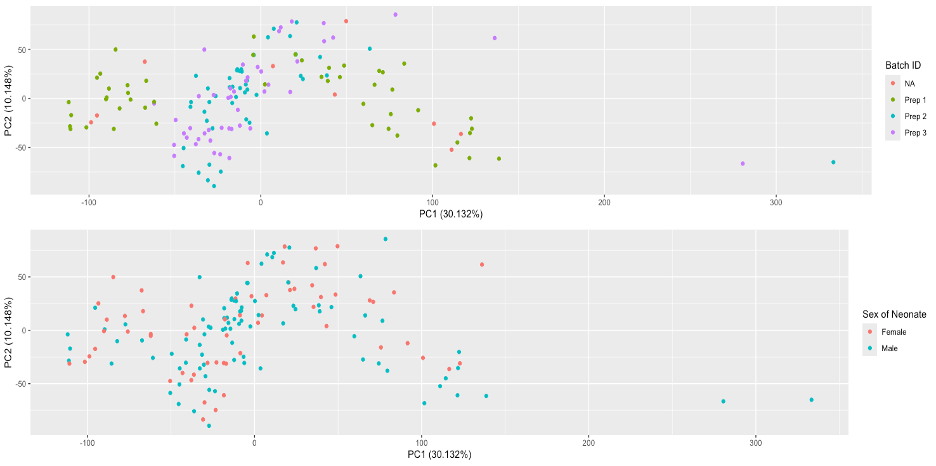

Supplement: 5 [file NIHMS2109826-supplement-5.png]

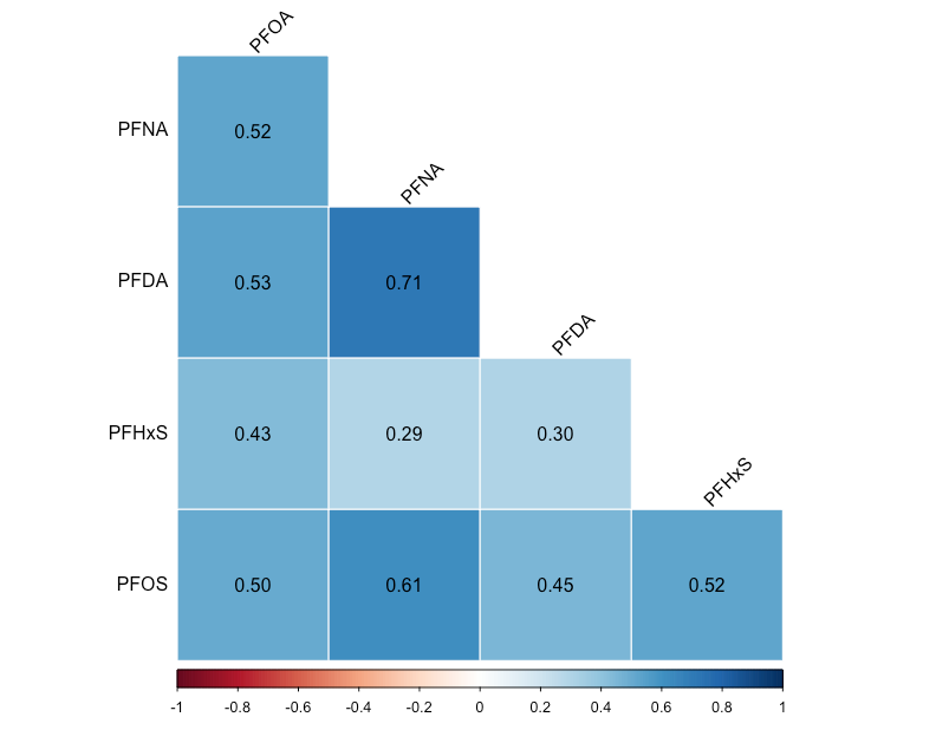

Supplement: 6 [file NIHMS2109826-supplement-6.png]
